# Effects of Traditional Chinese Medicine Rehabilitation Program on Knee Osteoarthritis in Aging Population: A Multicenter Randomized Controlled Trial

**DOI:** 10.1111/1756-185x.70398

**Published:** 2025-08-25

**Authors:** Weihong Zhong, Jiajia Ye, Daniel Kwasi Ahorsu, Xiaoqin Chen, Shanli Yang

**Affiliations:** ^1^ Department of Orthopedics Rehabilitation Hospital affiliated to Fujian University of Traditional Chinese Medicine Fuzhou Fujian China; ^2^ Fujian Key Laboratory of Rehabilitation Technology Fuzhou Fujian China; ^3^ Department of Rehabilitation Assessments Rehabilitation Hospital Affiliated to Fujian University of Traditional Chinese Medicine Fuzhou China; ^4^ Department of Special Education and Counseling The Education University of Hong Kong China; ^5^ Faculty of Acupuncture‐Moxibustion and Tuina Fujian Traditional Chinese Medicine University China; ^6^ Rehabilitation Hospital affiliated to Fujian University of Traditional Chinese Medicine Fuzhou Fujian China

**Keywords:** aging, knee, osteoarthritis, rehabilitation, traditional Chinese medicine

## Abstract

**Introduction:**

Knee osteoarthritis (KOA) is a prevalent degenerative disease that causes pain and disability in older individuals. This study aimed to examine the effects of the traditional Chinese medicine (TCM) rehabilitation program for aging populations with KOA.

**Methods:**

A total of 101 participants with KOA were randomly assigned to either a TCM rehabilitation group (*n* = 49) or a conventional physical therapy group (*n* = 52) with a 1:1 allocation ratio for this randomized controlled trial. Participants in the TCM group received acupuncture, massage, and South Shaolin exercise training for 4 weeks, with three sessions per week lasting 50 min per session. Participants in the control group received conventional physical therapies of equal duration and frequency.

**Results:**

Outcomes were Western Ontario and McMaster Universities Osteoarthritis Index (WOMAC), Visual Analogue Scale (VAS), Knee Outcome Survey Activities of Daily Living Scale, the 6‐min Walking test (6MWT), the Time Up and Go (TUG) Test, and the Stair‐climbing Test. Significant improvements were observed in the WOMAC, VAS, 6MWT, TUG, and Stair‐climbing test after a 4‐week TCM rehabilitation intervention (*p* < 0.05). The WOMAC and VAS were found to be decreased at the 4‐week follow‐up assessments compared to baseline scores (*p* < 0.05). Only the TUG test showed significant changes in the control group compared with the TCM rehabilitation group (*p* = 0.043) after 4 weeks post‐intervention.

**Conclusion:**

The TCM rehabilitation program improved knee function and reduced pain intensity in aging populations with knee osteoarthritis. Well‐designed randomized controlled trials with long‐term follow‐up assessments are needed to draw more definitive conclusions. Trial registration Chinese Clinical Trial Register ChiCTR2000033351, date of registration: May 29, 2020.

## 1 Introduction

Knee osteoarthritis (KOA) is a common joint disorder that leads to significant pain and disability, especially in the aging population worldwide. The initial damage of KOA is primarily to articular cartilage, with progressive deterioration in the membrane, subchondral bone, ligaments, joint capsule, and periarticular muscles in the later stage [1]. It is estimated that approximately 17% of the global population with ages ranging from 50 to 75 years have symptoms of KOA [2, 3]. The prevalence of KOA is around 13% in women and 10% in men among individuals aged 60 years and older. It increases with age and obesity, imposing a substantial health and socioeconomic burden [4].

Despite the significant impact of KOA, it has been observed that most older adults with KOA have insufficient treatments [3]. The current mainstream treatment for KOA is taking nonsteroidal anti‐inflammatory drugs (NSAIDs), but the affordability of NSAIDs remains a challenge for economically disadvantaged older adults [5]. Physical exercise has been shown to have beneficial effects for KOA, but its acceptance has not been widespread among the aging Chinese population due to cultural factors and the lack of culturally tailored exercise programs [6].

Traditional Chinese medicine (TCM) has been practiced for over 2000 years in China, with its practices primarily grounded in clinical experience rather than scientific evidence [7]. TCM therapies, including Chinese herbs, acupuncture, massage, Tai chi, Qigong, and moxibustion, are embraced by various age groups within the Chinese community, particularly among older adults due to their minimal side effects and perceived clinical effectiveness. A specialized training program combining acupuncture, massage, and South Shaolin exercise, developed by the Osteoarthritis Rehabilitation Research Center at the Fujian Rehabilitation Hospital in China, has been previously reported to demonstrate considerable clinical efficiency and safety [8]. However, the comparative data on the effectiveness of this TCM training program versus other conventional therapies remain limited. Thus, there is an urgent need to conduct an experimental study to examine the therapeutic effects of this training program among the aging Chinese population afflicted with KOA. Therefore, a multicenter randomized controlled trial study was proposed to assess the effects of a 4‐week TCM training program among the aging Chinese population with KOA. This study aimed to provide valuable insights into the efficacy of the TCM training program. Specifically, the objective was to examine whether there would be significant improvements in the Western Ontario and McMaster Universities Osteoarthritis Index (WOMAC) scale, Visual analogue scale (VAS), Time up and go test (TUG), Knee Outcome Survey Activities of Daily Living Scale (KOS‐ADLS) score, 6‐min walking test (6MWT), and Stair test after a 4‐week TCM rehabilitation program. The findings of this study may offer an evidence‐based alternative for the management of KOA. It is, therefore, hypothesized that the TCM rehabilitation program would enhance knee function and alleviate pain in individuals with knee osteoarthritis.

## 2 Methods and Materials

### 2.1 Study Design

This multicenter, randomized, single‐blind, two‐arm parallel assignment, controlled trial was approved by the Ethics Institutional Review Boards of Fujian Rehabilitation Hospital and Gansu Provincial Hospital of Chinese Medicine (Trial Registration No. ChiCTR2000033351). The study was carried out according to the Declaration of Helsinki, and the procedures and results were reported in accordance with the CONSORT checklist.

### 2.2 Participants

Participants were recruited through advertisements and referrals from their doctors at the Rehabilitation Hospitals (Fujian, China) and Gansu Provincial Hospital of Chinese Medicine (Gansu, China) from June 1, 2020 to April 2, 2022. Eligibility criteria included: (1) aged 50 to 80 years; (2) KOA diagnosis as per the *Diagnosis and Treatment of Osteoarthritis guidelines* [9]; (3) radiological diagnostic grade between 2 and 3 based on the Kellgren–Lawrence (K‐L) criteria [5]; and (4) voluntary participation with signed informed consent. Participants were excluded if they: (1) have comorbidities such as psoriasis, syphilitic neuropathy, Charcot's arthropathy, brown‐yellow disease, metabolic osteopathy, and acute trauma [8]; (2) have secondary or traumatic KOA; (3) have received intra‐articular steroid injection or joint replacement in the past 3 months; (4) have total knee replacement in the past year; (5) were unable to walk independently; and (6) were not able to communicate.

### 2.3 Sample Size Calculation

The sample size calculation for this RCT was based on the author's previous study [5]. The previous study found that the mean and standard deviation of WOMAC among the mind–body intervention group was 20.08 ± 9.95, and 30.16 ± 10.95 for the control group. The effect size measured by Cohen's d was calculated to be 0.45. A total of 124 participants were required to achieve sufficient statistical power (1‐*β* = 0.8). To account for a potential drop‐out rate (estimated to be 10%), a total of 138 participants were needed for the 2 groups.

### 2.4 Procedure

A qualified research assistant who received diagnostic training from a medical doctor explained the research and conducted an initial screening for eligibility. All eligible participants were randomly assigned to either the TCM rehabilitation group or the physiotherapy control group in a 1:1 ratio using computer‐generated numbers. Participants were informed about their group allocation (TCM intervention or physiotherapy) after baseline assessments through phone calls by a research assistant. Two blinded research assistants conducted all outcome assessments. All participants provided signed informed consent before participation. The protocol for this study has been published in the Journal of Annals of Palliative Medicine https://doi.org/10.21037/apm‐21‐1179.

### 2.5 Intervention Program and Control Condition

#### 2.5.1 Traditional Chinese Medicine (TCM) Rehabilitation Program for the Treatment Group

The TCM rehabilitation program comprised acupuncture, massage, and South Shaolin exercise. Selected acupoints on the affected side of the leg included calf nose (ST35), inner knee eye (EX‐LE4), Liangqiu (ST34), Xuehai (SP10), Weizhong (BL40), Zusanli (ST36), Yanglingquan (GB34), Yinlingquan (SP9), and Ashi points. The participants were in a supine position, and disposable needles (0.3 × 40 mm, Hautuo, Wuhan, China) were injected 10–25 mm into the skin by 2 professional TCM practitioners with the “Deqi” sensation confirmed by participants. A 15‐min massage was administered after acupuncture, followed by a 5‐min South Shaolin exercise performed 4 times a day, with a 5‐min interval between each session, over the course of 4 weeks [8].

#### 2.5.2 Conventional Physiotherapy Program for the Control Group

Participants in the control group received a comprehensive physiotherapy program based on the guidelines recommended by the International Osteoarthritis Research Society. This program comprised multiple therapeutic components: isokinetic training to improve muscle support around affected knee joints, aerobic exercises to boost cardiovascular health, dynamic balance exercises to reduce fall risk and enhance stability, and active range of motion exercises to promote joint flexibility. Low frequency electrotherapy at 10 Hz and 30 mA (No. XY‐K‐SISS‐A, Henan, China) was applied to relieve pain and reduce muscle tension, while neuromuscular training focused on enhancing muscle control and coordination. Each session lasted 40 min, conducted three times per week over a four‐week period, with two professional physiotherapists providing precise guidance and supervision to maximize therapeutic benefits [10].

### 2.6 Outcome Measures

Clinical assessments were conducted at baseline, 4‐week post‐intervention, and 4‐week follow‐up intervals. All these assessments were consistently conducted in the same rooms by 2 research assistants.

#### 2.6.1 Visual Analogue Scale (VAS)

The VAS was widely used for assessing pain severity. Participants were asked to indicate their subjective pain feeling on a 10‐cm line, with 0 representing “no pain” and 10 suggesting “unbearable pain.”

#### 2.6.2 Western Ontario and McMaster Universities Osteoarthritis Index (WOMAC) Scale

The WOMAC was used to assess participants' physical function (with 17 questions), pain (with 5 questions), and stiffness (with 2 questions). The score ranges from 0 to 96. A higher total score indicates more severe knee dysfunction.

#### 2.6.3 Knee Outcome Survey Activities of Daily Living Scale (KOS‐ADLS) Score

The KOS‐ADLS was a patient‐report scale used to assess the symptoms and functional limitations in daily activities for individuals with knee disorders [11]. It comprises 17 items, with lower scores indicating a greater degree of disability [12].

#### 2.6.4 Time Up and Go Test (TUG)

The TUG was a test that basically assesses mobility skills, including balance and agility. The time taken for a participant to rise from a chair, walk 3 m, turn around, walk back, and sit down is recorded in seconds. A longer test completion time indicates greater mobility impairments [13].

#### 2.6.5 Six‐ Minute Walking Test (6MWT)

The 6MWT was used to assess endurance and the ability to walk longer distances [13]. Participants were asked to walk as far as possible in 6 min. The distance covered was recorded in meters.

#### 2.6.6 Stair Test

The stair test was a way to assess fitness function. Participants were instructed to ascend and descend a flight of 9 stairs in their usual manner. The time taken to complete this task was measured and recorded in seconds [8].

### 2.7 Safety Measures

Participants were required to complete a form after each intervention session to document any adverse events, health status, and their participation throughout this study. The adherence and the incidence of adverse events were analyzed by the research team to determine the relationship with the intervention.

### 2.8 Data Analysis

The demographic data of baseline characteristics are presented using descriptive statistics. The Shapiro–Wilk test was used to assess the normality of the data. Individual outcome measures were assessed using Mixed Factor Analysis of Variance (Mixed factor ANOVA; group × time) to identify both interaction and main effects. If a significant interaction effect emerged, a separate subgroup analysis with post hoc pairwise comparison between timepoints with the Bonferroni adjustment was conducted. Also, independent t‐tests and paired sample t‐tests were used to analyze data on the 6‐min walking test (6MWT), Time Up and Go test (TUG), and Stair‐Climbing test (Stair) with their effect sizes (Cohen's d). An intention‐to‐treat (ITT) analysis with the last observation carried forward (LOCF) method was employed for missing data. The ITT analysis was performed only on participants who completed at least 70% of the total intervention sessions. All statistical analyses were performed using SPSS 25 (IBM, New York, U.S.) using an alpha level of 0.05 to indicate a significant level.

## 3 Results

**FIGURE 1:**
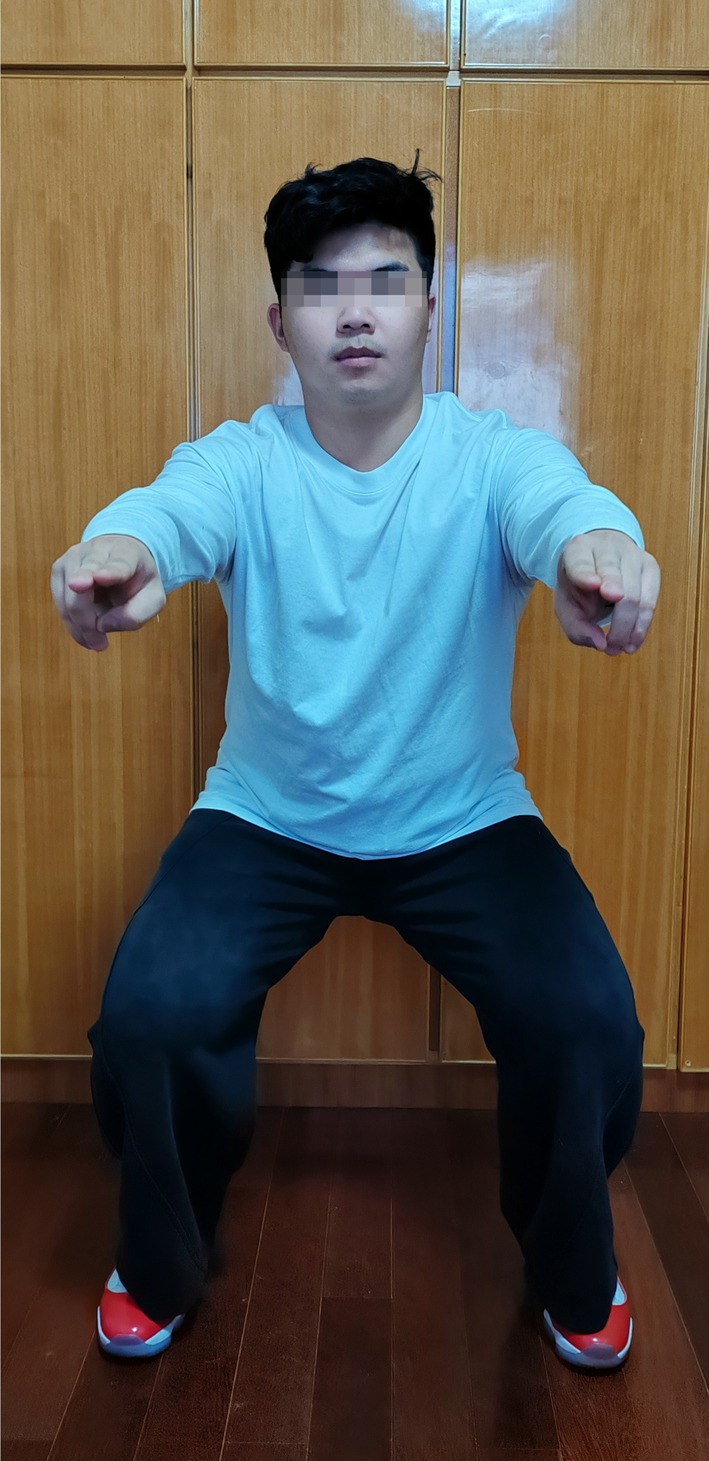
A posture demonstration of South Shaolin exercise.

The baseline characteristics of participants in both groups were comparable, as detailed in Table 1. The mean age was 66.24 years in the TCM rehabilitation group and 63.92 years in the control group, with females constituting approximately 90% of the participants. Initially, 113 participants were involved in this study. Nine of them did not attend the follow‐up assessment, and three of them did not receive the full course of treatment. Consequently, the data of 101 participants were ultimately analyzed, as illustrated in Figure 1. No adverse effects were reported throughout the study.

**TABLE 1:** Baseline value of all variables in two groups.

Characteristics	TCM (*n* = 49)	Con (*n* = 52)	*P*
Age, year	66.24 ± 10.79	63.92 ± 6.96	0.199
Gender: females, %	42, 93%	45, 87%	0.905
Height, cm	160.12 ± 6.15	161.87 ± 6.10	0.156
Weight, kg	61.86 ± 8.59	64.69 ± 7.50	0.080
Body mass index, kg/m2	24.13 ± 3.28	24.70 ± 2.61	0.337
Smoking (yes), %	3, 6%	4, 7%	0.759
Drinking (yes), %	3, 6%	4, 7%	0.759
Exercise (no), %	4, 8%	1, 2%	0.151
WOMAC	16.92 ± 7.76	20.15 ± 10.82	0.089
VAS	4.20 ± 1.66	4.54 ± 1.84	0.341
KOS‐ADLS, %	0.74 ± 0.14	0.74 ± 0.13	0.766
6MWT(m)	3.86 ± 0.86	3.95 ± 0.94	0.608
TUG(s)	10.46 ± 2.43	10.00 ± 1.81	0.303
Stair(s)	12.78 ± 3.13	14.12 ± 5.86	0.159

Abbreviations: 6MWT, the 6‐min walking test; KOS‐ADLS, Knee Outcome Survey Activities of Daily Living Scale; Stair, Stair‐climbing test; TCM, Traditional Chinese Medicine group; Con, Control group. TUG, Time up and go test; VAS, visual analogue scale; WOMAC, Western Ontario and McMaster Universities Osteoarthritis Index.

**FIGURE 2:**
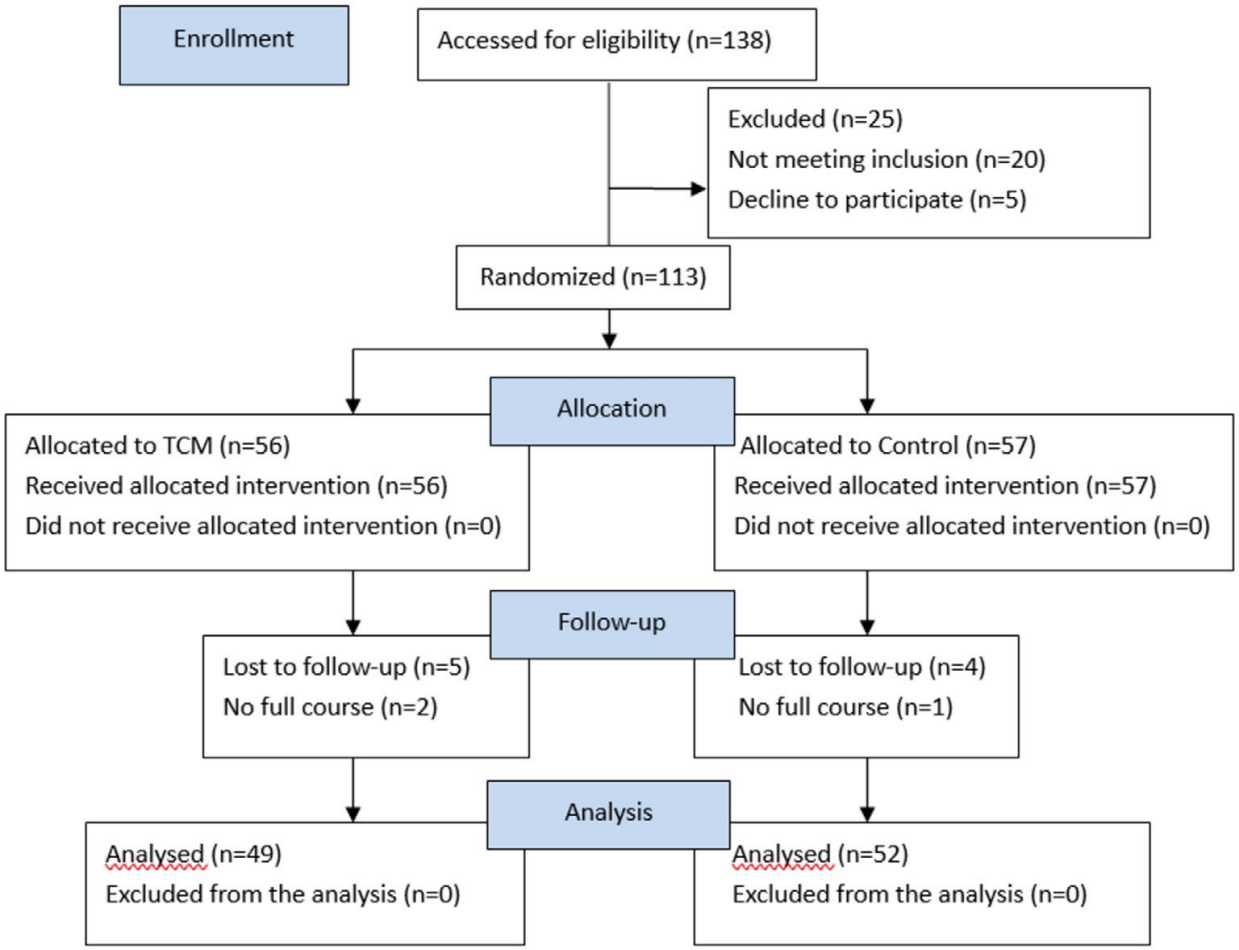
Flowchart of this study.

The WOMAC, VAS, and KOS‐ADLS assessments were conducted at three timepoints: baseline, 4 weeks post‐intervention, and at a 4‐week follow‐up (Tables 2 and 3) (Figure 2). Due to pandemic‐related restrictions, the 4‐week follow‐up assessments were conducted via phone. Participants completed the 6MWT, TUG, and Stair tests at baseline and 4 weeks post‐intervention. However, these assessments were not conducted at the 4‐week follow‐up due to the same restrictions. Significant time effects were observed for both WOMAC and VAS scores (*p* = 0.001) (Table 3). Additionally, significant within‐group differences were observed in the 6MWT, TUG, and Stair tests in the two groups between baseline and 4 weeks post‐intervention (*ps* < 0.05) (Tables 4 and 5). However, there were no significant differences between groups across all outcome measures except the TUG test (*p* = 0.043). The details are shown in (Tables 4, 5 and 6).

**TABLE 2:** Mean and SD performance value of WOMAC, VAS, and KOS‐ADLS.

Variable Group	Mean ± SD		Condition effect	Time effect	Condition × time effect		
	Week 4	Week 8	*p*	*p*	*F*	*p*	η^2^
WOMAC			0.224	0.001*	1.588	0.209	0.016
TCM	11.18 ± 7.19	7.10 ± 5.12					
Con	12.02 ± 9.39	8.33 ± 8.53					
VAS			0.318	0.001*	0.197	0.783	0.002
TCM	2.78 ± 1.25	2.07 ± 1.18					
Con	2.92 ± 1.41	2.29 ± 1.17					
KOS‐ADLS			0.676	0.346	0.065	0.848	0.001
TCM	77% ± 23.4	76% ± 25.2					
Con	75% ± 23.4	72% ± 23.6					

*Note:* Knee Outcome Survey Activities of Daily Living Scale.

Abbreviations: Con: Control group, TCM: Traditional Chinese Medicine group.

* Denotes a significant effect (*p* < 0.05).

**TABLE 3:** Within‐group differences for WOMAC, VAS, and KOS‐ADLS in two groups.

Variable		Pre vs. Week 4		Week 4 vs. Week 8		Pre vs. Week 8	
		TCM	Con	TCM	Con	TCM	Con
WOMAC	*p*	0.001*	0.001*	0.001*	0.001*	0.001*	0.001*
	95% CI	4.13 to 7.33	6.00 to 10.27	2.15 to 6.02	1.90 to 5.49	7.46 to 12.17	9.48 to 14,19
VAS	*p*	0.001*	0.001*	0.001*	0.001*	0.001*	0.001*
	95% CI	0.93 to 1.77	1.06 to 1.94	0.33 to 1.00	0.37 to 0.88	1.55 to 2.45	1.70 to 2.55
KOS‐ADLS	*p*	0.456	0.560	0.105	0.026*	0.907	0.757
	95% CI	−0.09 to 0.04	−0.09 to 0.05	−0.01 to 0.05	0.01 to 0.06	−0.07 to 0.07	−0.06 to 0.08

Abbreviations: Con, Control group; KOS‐ADLS, Knee Outcome Survey Activities of Daily Living Scale; TCM, Traditional Chinese Medicine group; VAS, visual analogue scale; WOMAC, Western Ontario and McMaster Universities Osteoarthritis Index.

* Denotes a significant effect (*p* < 0.05).

**TABLE 4:** The differences in 6MWT, TUG, and Stair between pre‐and post‐4 weeks intervention.

Variable	6MWT(m)				TUG(s)				Stair(s)			
	Baseline	Week 4	*p*	*d*	baseline	Week 4	*p*	*d*	baseline	Week 4	*p*	*d*
TCM	3.86 ± 0.86	4.15 ± 0.88	0.005*	−0.33	10.46 ± 2.43	4.76 ± 0.44	0.001*	3.27	12.78 ± 3.13	11.87 ± 3.07	0.005*	0.29
Con	3.95 ± 0.94	4.24 ± 0.93	0.005*	−0.31	10.00 ± 1.81	4.52 ± 0.65	0.001*	4.03	14.12 ± 5.86	12.60 ± 4.56	0.001*	0.29
*P*	0.608	0.647			0.303	0.043*			0.159	0.364		

Abbreviations: 6MWT, the 6‐min walking test; Con, Control group. Stair, Stair‐climbing test; CM, Traditional Chinese Medicine group; TUG, Time up and go test.

* Denotes a significant effect (*p* < 0.05).

**TABLE 5:** Between‐group differences for WOMAC, VAS, and KOA‐ADLS.

Variable		Week 4	Week 8
WOMAC	*P*	0.618	0.387
	95% CI	−4.15 to 2.48	−4.02 to 1.57
VAS	*P*	0.412	0.258
	95% CI	−0.68 to 0.41	−0.71 to 0.27
KOA‐ADLS	*P*	0.109	0.115
	95% CI	0.05 to −0.08	0.05 to −0.07

Abbreviations: KOS‐ADLS, Knee Outcome Survey Activities of Daily Living Scale; VAS, visual analogue scale; WOMAC, Western Ontario and McMaster Universities Osteoarthritis Index.

**TABLE 6:** Mean change and MCID for outcome variables.

Variables	Pre‐Week 4 Mean change (SD)	Week 4‐Week 8 Mean change (SD)	Pre‐Week 8 Mean change (SD)	MCID
**WOMAC** TCM Con	5.73(5.56) 8.13(7.65)	4.08(6.74) 3.69(6.45)	9.82(8.19) 11.83(8.47)	5.5^15^
**VAS** TCM Con	1.36(1.41) 1.49(1.50)	0.64(1.12) 0.64(0.89)	2.0(1.50) 2.13(1.43)	2.0^16^
**KOA‐ADLS** TCM Con	−2.54(23.64) −2.03(25.0)	2.13(9.01) 3.08(9.67)	−0.41(24.21) 1.04(24.17)	2.2^15^
**6MWT(m)** TCM Con	−25.69(58.52) −18.56(43.87)	‐‐‐‐—	‐‐‐‐—	61^17^
**TUG(s)** TCM Con	0.65(1.57) 0.75(1.44)	‐‐‐‐—	‐‐‐‐—	2.82^17^
**Stair** TCM Con	1.67(3.54) 1.32(2.39)	‐‐‐‐—	‐‐‐‐—	5.5^18^

Abbreviations: MCID, Minimal Clinically Important Difference; WOMAC, Western Ontario and McMaster Universities Osteoarthritis Index; VAS, visual analogue scale; KOS‐ADLS, Knee Outcome Survey Activities of Daily Living Scale; Stair, Stair‐climbing test; TUG, Time up and go test; TCM, Traditional Chinese Medicine group; Con, Control group.

## 4 Discussion

The present study examined the effects of a TCM rehabilitation program on physical functioning and pain‐related outcomes among Chinese aging populations with KOA. Results indicated that a 4‐week TCM rehabilitation program improved pain sensation, physical function, strength, mobility, endurance, and fitness function similar to those who went through a comprehensive physiotherapy program based on the guidelines recommended by the International Osteoarthritis Research Society. No significant between‐group differences were observed across all outcome measures except the TUG test.

Significant improvements in the WOMAC and VAS scores are consistent with the existing literature, suggesting that both TCM rehabilitation practices and conventional therapy can effectively reduce pain and improve physical functioning in patients with KOA [6, 7, 14, 15]. Similarly, the improvements in the 6MWT, TUG, and Stair Tests in the TCM rehabilitation group were aligned with previous findings, indicating that the TCM rehabilitation program significantly enhanced mobility, endurance, and fitness function similar to those who go through a comprehensive physiotherapy program [16]. However, those who went through a comprehensive physiotherapy program seem to have significant mobility skills compared to those who went through the TCM rehabilitation program.

Several factors contributed to understanding the present findings. Firstly, acupuncture, a key component of the TCM rehabilitation program, may facilitate the release of endogenous opioids and serotonin in the central nervous system, playing a vital role in analgesic and anti‐inflammatory effects [17, 18]. This led to improved joint function and reduced pain intensity, as reflected in WOMAC and VAS scores [5, 6].

Secondly, massage therapy, another important component of this TCM rehabilitation program, has been shown to have beneficial effects on blood circulation and lymphatic flow, contributing to reducing muscular tension and promoting relaxation [19, 20]. These physiological responses may increase circulation, facilitate the removal of inflammatory mediators, and increase the flow of nutrients to reduce pain intensity.

Thirdly, South Shaolin exercise, a form of Qigong that combines mind, body movement, and breathing techniques, has been associated with improvements in physical function and pain reduction [21]. This exercise emphasized the mind–body integration, which promoted muscle strength, endurance, and physical function [22]. These benefits may lead to better performance in functional tests such as the 6MWT, TUG, and Stair test. Moreover, the mediative component of this exercise also played a vital role in pain management.

The observed enhancements in pain relief, physical function, endurance, strength, balance, and overall fitness can be attributed to the multifaceted modalities of this Traditional Chinese Medicine (TCM) rehabilitation program. Specifically, acupuncture played a considerable role in diminishing pain and reducing inflammation. Massage therapy, on the other hand, enhanced blood circulation and facilitated muscle relaxation. Moreover, the incorporation of South Shaolin exercises strengthened muscles and improved balance, mobility, and fitness function. These benefits were reflected not only in pain and functional capacity but also in enhanced mobility, endurance, balance, and physical fitness.

Although these noticeable improvements were observed after the 4‐week TCM rehabilitation program, there was no significant enhancement in daily activities, as measured by the KOA‐ADLS. This observation aligned with prior studies [23], suggesting that daily activities involved a broad spectrum of motions that were not entirely covered by the general practices of acupuncture, massage, and South Shaolin exercise. Additionally, South Shaolin exercise was only practiced for 5 min. This may not be sufficient to significantly impact daily activities. Further studies might require more personalized or activity‐specific interventions to explain this finding.

Interestingly, the control group showed a significantly greater improvement in the Timed Up and Go (TUG) test compared to the TCM rehabilitation group. Chun et al. [24] reported that muscle strength was the primary modifiable factor associated with enhanced physical performance. This finding was supported by Qiu et al. [25] who observed that TUG performance improved more in the exercise group than in the general treatment group for patients with knee osteoarthritis (KOA). In this study, participants in the control group received isokinetic muscle training targeting the affected knee joints, which may have significantly improved knee muscle strength compared to the TCM group, who only performed static and semi‐squatting exercises (South Shaolin exercises). These TCM exercises may comparatively lack sufficient intensity to effectively strengthen the muscles surrounding the knee joints. Overall, conventional physical therapy perhaps has a more direct impact on muscle strength, balance, speed, and agility necessary for better TUG test outcomes compared to TCM rehabilitation interventions [26]. However, few studies have directly compared TCM rehabilitation programs with conventional physical therapy in KOA patients. Further research with specific outcome measures to confirm these findings is highly needed [14].

Despite the interesting findings of this study, several limitations should be noted. First, participants in the control group received conventional therapy for KOA, which may have minimized the observed differences between the TCM rehabilitation and control groups. Waitlist controls in future studies should be considered to better isolate the therapeutic effects of the TCM rehabilitation program. Second, the recruitment of participants exclusively from hospitals may limit the generalizability of the results. Expanding the study to include more medical centers and diverse community settings would enhance the applicability of the findings. Third, the majority of participants were female in this study. This may have introduced heterogeneity into the results. Therefore, the findings of this study should be interpreted with caution. Fourth, lockdown measures significantly shortened the data collection period, reducing the total number of participants. Finally, due to constraints related to the COVID‐19 pandemic, certain outcome measures could not be fully implemented as initially planned, as some participants were unable to complete specific assessments. Future studies are encouraged to include more comprehensive follow‐up assessments to evaluate the long‐term impacts of the TCM rehabilitation program.

## 5 Conclusion

This RCT demonstrated that the TCM rehabilitation program, comprising acupuncture, massage, and South Shaolin exercise, was effective in relieving pain intensity and enhancing physical function, endurance, strength, mobility, and fitness function among Chinese aging populations with KOA. It indicated that a 4‐week regimen involving 3 sessions per week with each session lasting 50 min can be designed for KOA patients to gain positive effects on the knee joint among the elderly with KOA.

## Author Contributions

**Weihong Zhong**, **Xiaoqin Chen**, and **Shanli Yang** conceived the experiment and collected data. **Jiajia Ye** and **Daniel Kwasi Ahorsu** analyzed the data and drafted the manuscript. **Jiajia Ye**, **Daniel Kwasi Ahorsu**, **Xiaoqin Chen**, and **Shanli Yang** critically reviewed and approved the final version of the manuscript. All authors contributed to the article and approved the submitted version.

## Conflicts of Interest

The authors declare no conflicts of interest.

## Data Availability

The data that support the findings of this study are available on request from the corresponding author. The data are not publicly available due to privacy or ethical restrictions.
